# Ensuring low-emission electricity purchasing requires a broader systems perspective

**DOI:** 10.1016/j.isci.2025.112349

**Published:** 2025-04-04

**Authors:** Lissy Langer, Kenneth Bruninx, Anders Bjørn, Lukas Barner, Julien Lavalley, Hadi Vatankhah Ghadim, Rasmus Bramstoft

**Affiliations:** 1 Department of Technology, Management and Economics, Technical University of Denmark, Kgs. Lyngby, Denmark; 2 Department of Technology, Policy and Management, TU Delft, Delft, the Netherlands; 3 Department of Environmental and Resource Engineering, Technical University of Denmark, Kgs. Lyngby, Denmark; 4 Workgroup for Economic and Infrastructure Policy (WIP), Department of Economics and Management, Technische Universität Berlin, Berlin, Germany; 5 Department of Energy, Transportation, Environment, German Institute for Economic Research, Berlin, Germany; 6 Electricity Maps, Copenhagen, Denmark; 7 Sustainable Energy Research Group (SERG), Department of Civil and Natural Resources Engineering, University of Canterbury, Christchurch, New Zealand

**Keywords:** Molecular biology, Cell biology, Cancer

## Abstract

Introducing electricity purchase conditions in renewable fuel regulations and carbon accounting is a controversial issue in the US and Europe. We argue that their impact must be assessed considering demand flexibility, local grid conditions, and overlapping policy instruments such as emissions trading schemes and renewable portfolio standards. The introduction of more stringent requirements has a significant impact on companies' reported progress in reducing indirect emissions. In addition, we currently lack reliable carbon intensity data with sufficient temporal and spatial granularity outside the US and Europe. Assessing hydrogen imports should consider strategic behavior. In summary, a broader systems perspective is needed to analyze the impact of electricity purchase conditions on markets, prices, and emissions, integrating voluntary actions to decarbonize hard-to-abate sectors under increasing renewable energy shares. This will have implications not only for the (re)design and integration of certificate markets for renewable electricity but also for renewable fuels and carbon.

## Introduction

Electricity demand is expected to grow significantly, driven by decarbonization in the heating, industrial, and transportation sectors, as well as a growing demand from data centers and hydrogen production via electrolysis. Large amounts of renewable/clean hydrogen and its derivatives are being discussed to decarbonize hard-to-abate sectors such as heavy industry, aviation, and shipping. However, producing these fuels requires significant amounts of renewable/clean electricity, which is also needed to decarbonize other sectors.

Several studies have shown that the electrification of road transport and heating results in lower emissions than their conventional alternatives,^1^^,^^2^^,^^3^ the case for increased electricity demand for electrolysis and data centers is more complex. Without clear standards, the additional electricity demand in these sectors could increase greenhouse gas emissions.

Previous studies of 24/7 renewable/clean electricity purchasing for data centers and renewable hydrogen production have shown that demand-side flexibility plays a critical role in reducing both costs and emissions while also contributing to increased overall social welfare.^4^^,^^5^^,^^6^^,^^7^ To capture these benefits, policy interventions such as support schemes should be designed carefully, considering their impact on renewable hydrogen production profiles. For example, subsidies that consist of production tax credits, such as the US IRA,^8^ may inadvertently encourage the continuous baseload operation of electrolysis and increase system emissions.

The European Commission has defined three pillars^9^ for the production of renewable hydrogen to achieve at least 70% reduction in emissions compared to conventional production via steam methane reforming using unabated natural gas.^10^ These conditions for the purchase of electricity to produce renewable fuels of non-biological origin (RFNBOs) include: 1) an additionality proxy that aims to ensure that the renewable electricity used to produce the hydrogen would not have been produced otherwise (from 2028), 2) geographic deliverability of the renewable electricity to the hydrogen production site, and 3) hourly temporal matching of the renewable electricity and the hydrogen production (from 2030). In the US, similar rules and strict emission thresholds have been implemented for the maximum (uncapped) production tax credit of $3 per kg of hydrogen produced under the IRA 45V rules.^8^^,^^11^^,^^12^ In February 2024, the EU-wide pilot auction for RFNBO hydrogen resulted in six successful projects receiving €694 million, with bids ranging from €0.37–0.48 per kg of hydrogen. The second auction closed in February 2025 with 61 bids to be evaluated by the end of May.^13^

In addition, similar rules for corporate electricity purchases are being discussed in the ongoing revision of the Greenhouse Gas Protocol.^14^^,^^15^^,^^16^ The GHG Protocol defines the carbon accounting rules that underpin companies’ emission reduction and science-based targets.^17^ The original GHG Protocol for the accounting of emissions from purchased and consumed electricity, heat, and cooling (known as “scope 2”) dates to the 1990s and was last amended in 2015. The current market-based accounting rules allow companies to “neutralize” corporate emissions by purchasing “unbundled” renewable energy certificates (RECs), i.e., not in combination with electricity, matched with consumption over the whole year, from the same market, such as all of the US or Europe. For example, they allow a Polish data center running 24/7 in a carbon-intensive grid such as Poland to offset emissions by buying RECs from a 30-year-old hydropower plant in Iceland (an island 2600 km away) or from a solar PV plant in Spain (mainly producing in summer and during the day). Studies have warned that this accounting method may not result in additional renewable generation and lower system emissions and may allow companies to understate their emissions.^18^^,^^19^^,^^20^^,^^21^

Several studies using techno-economic energy system models have analyzed the system impacts of the proposed electricity purchase regulations for hydrogen production in the EU^4^^,^^22^ and the US^5^^,^^6^ and for corporate electricity procurement,^7^^,^^23^ focusing primarily on the temporal matching aspects of the proposed rules and their impacts on system costs and emissions.^24^

## Results

**Figure 1:**
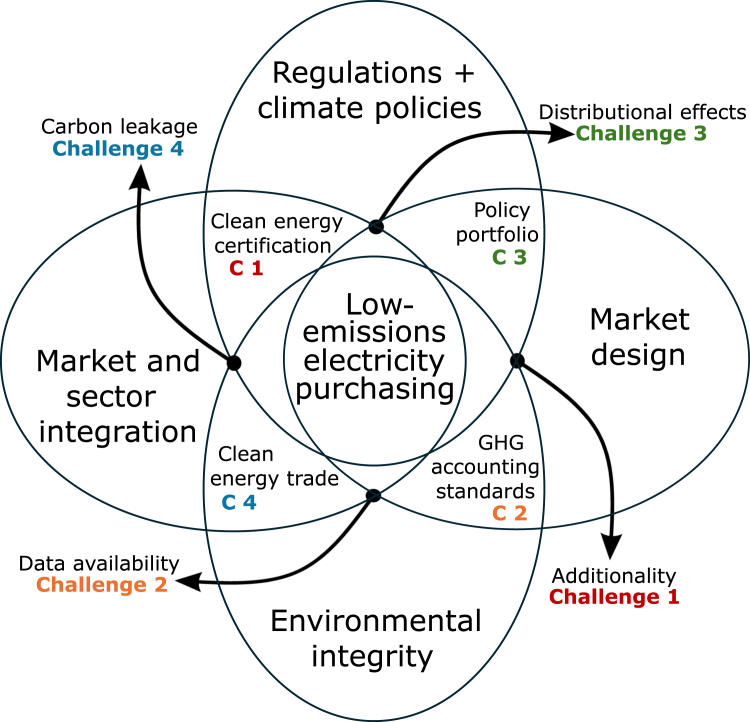
Challenges of low-emissions electricity purchasing in the broader systems perspective

We argue that a broader systems perspective is required to analyze the impact of electricity purchase conditions on markets, prices, and emissions, to decarbonize hard-to-abate sectors, and to integrate voluntary corporate action in an energy system with increasing shares of renewable energy sources. Our analysis focuses on the European market, but we also offer policy recommendations for other regions. In the following, we identify and discuss four key challenges to fully understand and mitigate system impacts (Figure 1).

(1) REC market designs need to evolve to better align electricity and hydrogen regulations, reflecting their interdependence. A measurable and enforceable definition of what constitutes additional renewable generation is needed.
(2) Carbon accounting standards must ensure that companies’ reported progress toward their emissions reduction targets is genuine. More granular data on emission intensities is critical, as current limitations prevent comprehensive global analysis.
(3) The interaction of regulations with the cap-and-trade mechanisms, such as the EU ETS, and the need for emissions and carbon pricing safeguards should be further explored.
(4) The role of hydrogen imports, their certification under the carbon border adjustment mechanism (CBAM), and their impact on prices should be investigated.

### Challenge 1. Clean energy certification and additionality

Current market-based accounting rules allow companies to purchase unbundled renewable energy certificates (RECs) from a wide geographic area and match them within the same year to offset their emissions. The EU’s hydrogen regulation applies stricter rules, requiring certificates to be purchased through a PPA (“bundled” with electricity), from the same bidding zone (national or sub-national) and within the same hour (from 2030). Exceptions are foreseen for low carbon (<18 g CO_2_/MJ) or high renewable (>90%) power systems.^9^

**Figure 2:**
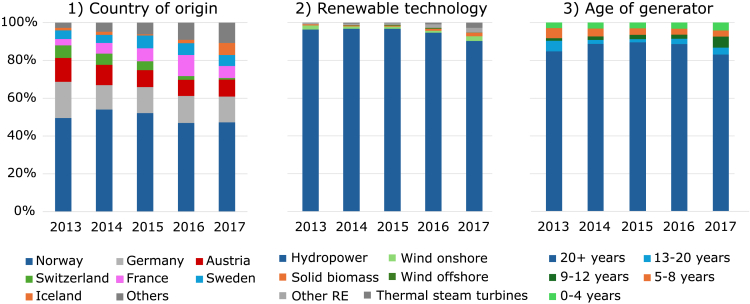
Guarantees of Origin (GOs) canceled in Germany 2013–2017^63^

Currently, utilities must purchase RECs (also known as ‘Guarantees of Origin’ (GOs) in Europe) for their renewable energy products. As an example, Figure 2 shows the RECs canceled in Germany from 2013 to 2017. Most of the RECs are from a) Norway (47% in 2017), b) hydropower (90% in 2017), and c) generators older than 20 years (83% in 2017). This suggests that existing plants capture windfall profits and demonstrates the limited impact of RECs on renewable energy investments. These accounting practices make it difficult for customers to distinguish between companies that have invested in their own local renewable generation and those that have purchased certificates from generators regardless of physical deliverability.

**Figure 3:**
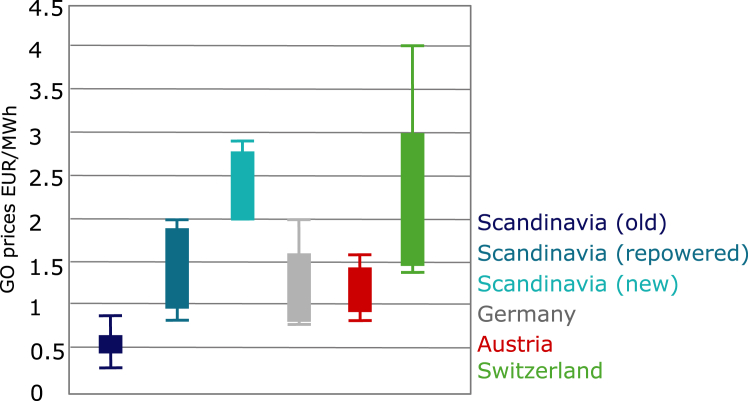
Price range of GOs in Europe in EUR/MWh based on interviews conducted in 2018^63^

In addition, annually matched RECs have historically resulted in low certificate prices (<€3 per MWh) compared to the average wholesale electricity price in Germany in 2024 of around €70 per MWh^25^ (Figure 3). This supplementary revenue is unlikely to be sufficient to incentivize additional investment in renewable energy.^26^^,^^27^ However, hourly matching and conditions to increase additionality could lead to higher prices with increased diurnal and seasonal variability.^28^ So far, however, one must rely on interview data as the Association of Issuing Bodies (AIB) in Europe only provides monthly trade values and no prices.

**Figure 4:**
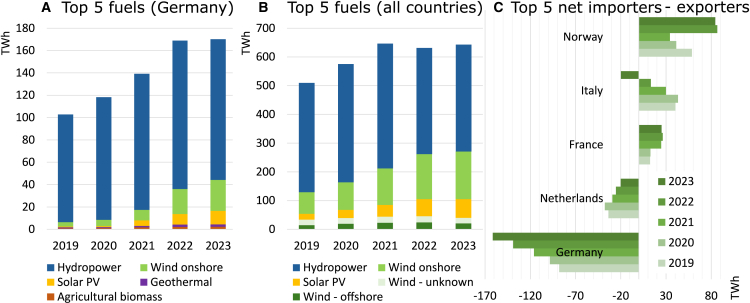
European GO cancellations and trade using AIB statistics 2019–2023 (A) Top 5 fuels used in RECs canceled in Germany, (B) Overall, and (C) Top 5 net importers and exporters.^29^

In recent years, Norway has continued to dominate the European REC market, while Germany remains the largest net importer of RECs (Figure 4C). Hydropower remains Germany’s and Europe’s largest source of RECs (Figures 4A and 4B). Unfortunately, the current statistics of the AIB do not include the origin-destination of REC transfers, the age of generators, or any REC prices.^29^ Although recent price data is not publicly available, a report from the UK based on interviews suggests that REC prices may have risen from around £0.20 per MWh before £2019 to £4−7 per MWh.^30^

Energy system models can help to determine the impact on REC prices, additional renewable energy, and system emissions of different market designs compared to a counterfactual without a REC market.^24^ These analyses could also consider the combined effects of REC purchases for hydrogen production, carbon accounting, and national renewable energy penetration targets. However, to quantify the impact on system emissions, it is necessary to carefully specify the technical, policy, and modeling assumptions that define the counterfactual.^24^ In practice, standards must be developed for the integration of certificate markets for renewable electricity, fuels, gases, and carbon.^31^

In addition, a clear definition of *additionality* is needed to ensure that renewable generation is truly additional compared to a counterfactual scenario without the policy interventions and does not simply cannibalize the decarbonization of other sectors. While some modeling studies enforce additionality to derive low-cost, low-emission cases,^4^^,^^24^ in practice, additionality is more difficult to verify because it is impossible to compare against a counterfactual scenario. However, so-called “additionality tests” have been proposed to ensure the integrity of market mechanisms for projects and policies.^16^^,^^32^ Four types have previously been used in the context of scope 2 guidance^33^: 1) *legal, regulatory, or institutional tests*, which check that the outcome is not required anyway, 2) *investment tests*, which check the financial specifics of the project, the duration of the contract, or that the project has not received government subsidies, 3) *timing tests*, which require that the project is ‘new’, and 4) *positive lists*, which explicitly define project types that are considered additional. They could be enforced on long-term contracts for RECs^34^ or power purchase agreements (PPA).^35^ Energy system studies could be extended to include these tests, also assessing risks and bankability for representative projects.

In addition, RECs could be established as statutory instruments with purchase obligations not only for renewable fuel producers and voluntary actors but also for EU countries to meet their renewable energy penetration targets in final energy consumption defined in their National Energy and Climate Plans (NECPs). These GOs, sometimes discussed as “GO+,” would thereby integrate the voluntary and compliance market and prevent double-counting.^31^

### Challenge 2. GHG accounting standards and data availability

Over the past two years, regulatory climate disclosure instruments have emerged around the world (see Table 1), incorporating to a significant extent the scope 2 standards and guidelines of the GHG Protocol.^36^ Companies are increasingly required to report on the environmental risks they face and how their actions affect the environment.^37^

**Table 1:** A selection of regulatory climate-related disclosure rules globally^36^

Program	International Sustainability Standards Board (ISSB)	EU Corporate Sustainability Reporting Directive (CSRD)	United States Securities and Exchange Commission (SEC)	California Air Resources Board (CARB)
Standard name	IFRS S2 Climate-Related Disclosures	European Sustainability Reporting Standards	The Enhancement and Standardization of Climate-Related Disclosures for Investors	Climate Corporate Data Accountability Act
Disclosure type	Voluntary; Mandatory when implemented at jurisdictional level (Australia, Canada, Japan)	Mandatory for companies in the EU with >1000 employees or turnover >50 MEUR or balance sheet >25 MEUR^64^	Mandatory for companies publicly traded in the US	Companies operating in California with over $1 billion in annual revenue
Differences to GHG Protocol’s scope 2	Requires only a location-based method	None (dual reporting required)	Dual reporting not required (method choice)	TBD
Uptake	∼100,000–130,000 companies globally	∼10,000 companies in EU and EU subsidiaries	∼4,000 companies	∼5,400 companies
Effective date	January 1, 2024	January 1, 2024 (or later, depending on company size)	January 1, 2025	January 1, 2025

However, Current GHG Protocol rules do not ensure accurate scope 2 emissions accounting.^38^ Bjørn et al. (2024)^39^ find that stricter accounting rules, closer to the “three pillars” of the EU RFNBO regulation, would render large fractions of the RECs purchased by companies today non-compliant, thereby increasing companies’ reported scope 2 emissions and worsening their performance against GHG reduction targets. Using company-level data on electricity consumption, REC purchases, and reported scope 2 emissions, the study analyzes the impact of three proposed accounting rules: 1) requiring geographic deliverability, 2) limiting the age of generators, and 3) requiring PPAs.

Results show that the 206 sample companies have increased their purchases of RECs over time, including RECs that would be invalid under stricter accounting rules. In 2022, about 56% of their REC purchases did not meet all three restrictions. Therefore, introducing stricter rules would reduce the apparent combined decarbonization achieved by the sample companies between their target base years and 2022 from 21% to 17%. This reduces the share of sample companies on track to meet their climate targets under all three accounting restrictions. However, a few companies have been moving to RECs that comply with all accounting restrictions, resulting in improved target performance for these companies. These results highlight the real-world implications of potential accounting restrictions in revising the GHG Protocol and various renewable fuel standards.

In addition, companies rely on emission factors to report their scope 2 emissions or calculate the carbon intensity of renewable hydrogen. These emission factors represent the local electricity grid mix or the residual grid mix without the RECs that companies have accounted for.

However, the current reporting methodology only suggests the use of time-delayed, annual average emission factors per country.^10^ This is likely because regularly updated emission factors specific to production modes, fuels, and electricity grids are difficult to obtain. This lack of availability affects the accuracy of emission calculations for hydrogen produced with grid electricity. Real-time electricity data is readily available in the US and Europe through EIA^40^ and ENTSO-E.^41^ However, outside of these regions, real-time data for hourly electricity generation and exchange is rarely available, and when it is available, it is scattered across multiple data sources. Companies such as Electricity Maps (https://www.electricitymaps.com/) but also the IEA (https://www.iea.org/data-and-statistics/data-tools/real-time-electricity-tracker) are collecting available data worldwide and developing estimation models to fill the gap with hourly granular data. Data availability (free of charge) is being discussed in the revision of the GHG Protocol. Possible solutions include transitional exemptions for some regions and a central repository of data sources for carbon accounting professionals.^42^

In addition, research is underway in Europe and the US to combine production and emissions data at the plant level to calculate more granular emission factors. However, these methodologies rely on data that are only available in some regions and face challenges such as electricity trading and the heat/electricity allocation for combined heat and power plants.^43^

Initiatives to increase the availability of open data in all regions of the world for electricity generation, exchanges, and emission factors should be encouraged and incentivized. These will be needed to ensure the comparability of emissions for clean hydrogen imports and corporate scope 2 accounting under more granular accounting rules.

### Challenge 3. Policy portfolios and distributional effects

In the public debate, the strict rules on electricity procurement for electrolytic hydrogen production (additionality, deliverability, and temporal matching) have been motivated primarily by fears of increasing CO_2_ emissions. However, the interaction of these rules with other policy instruments, such as the European Emission Trading System (EU ETS), is often not considered.

In Europe, EU ETS imposes a binding, annually decreasing cap on emissions from the electricity sector, energy-intensive industry, intra-EU aviation, and now also large ships sailing from or to European ports.^44^ It is enforced through tradeable emission allowances and prioritizes abatement in the sectors where it can be done at the lowest cost. Since the EU ETS covers the emissions from both fossil fuel and electricity-based hydrogen production, the strict requirements on renewable hydrogen do not seem well motivated as cumulative emissions remain unaffected, regardless of the hydrogen production route.^45^ The USA’s IRA 45V regulation acknowledges this interaction with cap-and-trade systems, exempting hydrogen producers from the additionality requirement in US states with “robust GHG emissions caps paired with clean electricity standards or renewable portfolio standards.”^12^ Currently, Washington’s and California’s emission trading systems and complementary renewable energy policies meet the criteria. In the US, however, the voluntary and compliance markets compete for RECs, unlike the EU, where the National Energy and Climate Plans (NECPs) account for all national renewable energy production (not consumption, so exports are included) without requiring the purchase of RECs.

Without strict electricity procurement rules for hydrogen production, the added pressure of a significant increase in electricity demand driven by ambitious hydrogen targets and electrification, combined with a declining emissions cap, could lead to a rise in European CO_2_ prices. This could threaten the competitiveness of the European industry, undermine the stability of the EU ETS as a whole, and cause unintended distributional effects.^46^^,^^47^ In addition, since 2019, the market stability reserve (MSR) has adjusted the effective emissions cap based on the cumulative difference between the supply of and demand for emission allowances. Therefore, any overlapping policy, such as introducing more wind or solar energy into the electricity system or enforcing hydrogen targets, that affects the demand for emission allowances may lead to changes in cumulative emissions from sectors covered by EU ETS.^48^ As a result, emissions may decrease due to stricter electricity purchase conditions, but these policies may, in theory, also backfire and increase emissions under the EU ETS.

However, as Europe must rapidly expand its renewable electricity supply to meet its stringent emission reduction targets for the sectors covered by the ETS (−62% w.r.t. 2005 by 2030, net-zero by 2050), the impact of (the absence of) strict electricity procurement rules on consequential emissions can be expected to be limited. This follows from the assumption that 1) the majority of electrolyzers will come online near and after 2030,^49^ 2) the European electricity system will be sufficiently decarbonized by then, and 3) flexible hydrogen production will avoid hours of high carbon intensity and electricity prices. Under these conditions, the consequential emissions associated with electrolytic hydrogen production will be limited, regardless of the stringency of electricity purchase requirements. Similarly, the impact on carbon prices would be limited.

This reasoning assumes electrolyzers are exposed to electricity prices, resulting in efficient siting, sizing, and dispatch decisions. Electrolyzers would be located in low-price, high-RES market zones and would not be dispatched during high-price, low-RES periods.^4^ Poorly designed support mechanisms or overly large market zones can distort this price signal,^50^ thereby increasing consequential emissions and having unpredictable impacts on cumulative emissions and carbon prices under the EU ETS.^48^ In other parts of the world with a lower expected renewable energy penetration and/or without a cap-and-trade system, the impact on cumulative emissions and/or carbon prices may be more pronounced.

### Challenge 4. International hydrogen trade and carbon leakage

The REPowerEU plan targets 10 Mt of domestic hydrogen production and 10 Mt of imports in 2030,^51^ reflecting the limited renewable resources available for hydrogen production. Also, at the national level, Germany’s Hydrogen Strategy targets 50−70% of demand to be imported by 2030.^52^^,^^53^ This corresponds to 45−90 TWh of imports or about 10% of the country’s current natural gas consumption. Other fossil fuel-importing countries outside the EU have similar ambitions, such as Japan.

If hydrogen becomes an internationally traded commodity, these markets will likely behave similarly to other energy commodities, such as oil and natural gas today. They are dominated by a few large firms with the ability to influence prices strategically,^54^^,^^55^ typically modeled as Nash-Cournot equilibria and other games.^56^^,^^57^^,^^58^

To date, policy-relevant studies have focused on the techno-economic costs of hydrogen production or individual trade routes rather than on market-based prices that might emerge.^59^^,^^60^^,^^61^ This observation includes energy system models used to investigate hydrogen procurement in various studies, which implicitly neglect the possibility of markups from strategic behavior. The first results from modeling imperfectly competitive global hydrogen markets indicate that Central Western Europe and some Asian countries, such as Japan or South Korea, would be most affected by the strategic behavior of hydrogen exporters.^62^

However, distortions from such behavior of exporters and domestic producers have not been adequately considered in the context of studies for the procurement of renewable/clean hydrogen. In particular, the effects of market power under varying electricity purchase conditions, such as those required to avoid carbon taxes on imported hydrogen under the carbon border adjustment mechanisms (CBAM) and the associated phase-out of the allocation of free allowances under the EU ETS, are poorly understood.

Given the uncertainty involved, understanding the effects of stochasticity, risk aversion, and strategic deterrence in the wider context of electricity purchasing will be of further interest to future research.

## Discussion

Electricity purchase conditions are currently being implemented, proposed, and discussed to support the decarbonization of renewable hydrogen production in the US and Europe and to ensure accurate corporate scope 2 emissions accounting. The common denominators show that the regulation, accounting, and certification of renewable electricity, fuels, gases, and carbon must increasingly be analyzed in an integrated manner from a broader systems perspective to understand and mitigate cumulative system impacts.

First, the impact of the three pillars – additionality, deliverability, and temporal matching - must be analyzed, taking into account demand flexibility and local grid characteristics. This must include the full policy portfolio, including emissions trading schemes, renewable portfolio standards, and national renewable energy penetration targets. In addition, all emerging certificate markets for renewable electricity, fuels, gases, and carbon must be integrated.

Second, the analysis must integrate compliance and voluntary markets, such as companies’ carbon emission reductions and progress toward their science-based targets. However, outside the US and Europe, the lack of reliable emission factors could significantly affect the accuracy of calculated emissions, suggesting the need for global open data initiatives and new methodologies.

Finally, hydrogen imports and the global hydrogen market need further study, given some players' potential market power and the resulting effects on cumulative system emissions.

Overall, the impact of electricity purchase conditions on markets, prices, and emissions seems to offer interesting insights for decarbonizing hard-to-abate sectors in an electricity system with higher shares of renewables. Certificate markets not only for renewable electricity but also for renewable fuels, gases, and carbon need to be further integrated to ensure the environmental integrity of the overall system.

## Acknowledgments

This article is an outcome of the Workshop on the Policy Context and Emissions Impact of Hydrogen and Power Purchase Conditions held on the 4th of July 2024 at the Technical University of Denmark. The workshop was supported by the Danish Energy Research Network ENERforsk and the NordNET project. This project has received funding from the European Union’s Horizon 2020 research and innovation programme under the Marie Skłodowska-Curie grant agreement no. 899987 and from Nordic Energy Research under the project Nord_H2ub (grant number 149089). Vatankhah Ghadim acknowledges funding from the Catalyst: Strategic Fund, administered by the New Zealand Ministry of Business Innovation and Employment, and the 10.13039/501100002347German Federal Ministry of Education and Research (BMBF) (grant number 03SF0690) for supporting the HINT project (New Zealand-German Platform for Green Hydrogen Integration). Lukas Barner acknowledges funding from the German Ministry for Education and Research (BMBF) for the MINDSET_Clean_H2 project (grant no. 03SF0780A).

## Author contributions

L.L.: conceptualization, data curation, visualization, and writing – original draft and review and editing; K.B.: conceptualization and writing – original draft and review and editing; A.B.: writing – original draft and review and editing; L.B.: writing – original draft and review and editing; J.L.: writing – original draft; H.V.G.: writing – review and editing; R.B.: conceptualization, writing – review and editing.

## Declaration of interests

The authors declare no competing interests.

## References

[1] IEA (2024). Global EV Outlook. https://www.iea.org/reports/global-ev-outlook-2024.

[2] IEA (2022). The Future of Heat Pumps. https://www.iea.org/reports/the-future-of-heat-pumps.

[3] Rosenow J. (2024). A meta-review of 54 studies on hydrogen heating. Cell Rep. Sustain..

[4] Zeyen E., Riepin I., Brown T. (2024). Temporal regulation of renewable supply for electrolytic hydrogen. Environ. Res. Lett..

[5] Ricks W., Xu Q., Jenkins J.D. (2023). Minimizing emissions from grid-based hydrogen production in the United States. Environ. Res. Lett..

[6] Giovanniello M.A., Cybulsky A.N., Schittekatte T., Mallapragada D.S. (2024). The influence of additionality and time-matching requirements on the emissions from grid-connected hydrogen production. Nat. Energy.

[7] Xu Q., Ricks W., Manocha A., Patankar N., Jenkins J.D. (2024). System-level impacts of voluntary carbon-free electricity procurement strategies. Joule.

[8] US Department of the Treasury (2023). Section 45V Credit for Production of Clean Hydrogen; Section 48(a)(15) Election To Treat Clean Hydrogen Production Facilities as Energy Property. https://www.federalregister.gov/d/2023-28359.

[9] European Commission (2023).

[10] European Commission (2023).

[11] Credit for Production of Clean Hydrogen and Energy Credit (2025). Federal Register. https://www.federalregister.gov/documents/2025/01/10/2024-31513/credit-for-production-of-clean-hydrogen-and-energy-credit.

[12] (2025). U.S. Department of the Treasury Releases Final Rules for Clean Hydrogen Production Tax Credit. https://home.treasury.gov/news/press-releases/jy2768.

[13] European Commission (2024).

[14] GHG Protocol (2022). Survey on Need for GHG Protocol Corporate Standards and Guidance Updates. https://ghgprotocol.org/survey-need-ghg-protocol-corporate-standards-and-guidance-updates?utm_medium=email&utm_source=publication&utm_campaign=ghgprotocol.

[15] GHG Protocol (2023). GHG Protocol Standards Update Process: Topline Findings From Scope 2 Feedback. https://ghgprotocol.org/sites/default/files/2023-05/Topline%20Findings%20from%20Scope%202%20Feedback%20Webinar_GHG%20Protocol_05.02.2023.pdf.

[16] GHG Protocol (2015). The GHG Protocol for Project Accounting. https://ghgprotocol.org/sites/default/files/standards/ghg_project_accounting.pdf.

[17] Science Based Targets initiative (2024). SBTi Corporate Net-Zero Standard. https://sciencebasedtargets.org/resources/files/Net-Zero-Standard.pdf.

[18] Bjørn A., Lloyd S.M., Brander M., Matthews H.D. (2022). Renewable energy certificates allow companies to overstate their emission reductions. Nat. Clim. Chang..

[19] Brander M., Gillenwater M., Ascui F. (2018). Creative accounting: A critical perspective on the market-based method for reporting purchased electricity (scope 2) emissions. Energy Policy.

[20] Elgin B., Rangarajan S. (2022).

[21] Rathi A., White N. (2024). How Tech Companies Are Obscuring AI’s Real Carbon Footprint. http://Bloomberg.com.

[22] Brauer J., Villavicencio M., Trüby J. (2022). Green Hydrogen – How Grey Can It Be?. https://hdl.handle.net/1814/74850.

[23] Riepin I., Brown T. (2024). On the means, costs, and system-level impacts of 24/7 carbon-free energy procurement. Energy Strategy Rev..

[24] Langer L., Brander M., Lloyd S.M., Keles D., Matthews H.D., Bjørn A. (2024). Does the purchase of voluntary renewable energy certificates lead to emission reductions? A review of studies quantifying the impact. J. Clean. Prod..

[25] Statista (2024). Electricity price Europe 2024 monthly. https://www.statista.com/statistics/1267500/eu-monthly-wholesale-electricity-price-country/.

[26] Gillenwater M., Lu X., Fischlein M. (2014). Additionality of wind energy investments in the U.S. voluntary green power market. Renew. Energy.

[27] Gillenwater M. (2013). Probabilistic decision model of wind power investment and influence of green power market. Energy Policy.

[28] Scholta H.F., Blaschke M.J. (2024). Shedding Light on Green Claims: The Impact of a Closer Temporal Alignment of Supply and Demand in Voluntary Green Electricity Markets. https://ceepr.mit.edu/wp-content/uploads/2024/06/MIT-CEEPR-WP-2024-08.pdf.

[29] Association of Issuing Bodies (2024). AIB Statistics. https://www.aib-net.org/facts/market-information/statistics/activity-statistics.

[30] Cornwall Insight (2023). REGOs and Decarbonisation. https://www.cornwall-insight.com/files/ovo-energy-ltd-regos-and-decarbonisation-af447adc.pdf.

[31] Pototschnig A., Conti I. (2023). *Law in the EU’s Circular Energy System: Biofuel, Biowaste and Biogas* (Lucila de Almeida and Josephine van Zeben.

[32] Michaelowa A., Hermwille L., Obergassel W., Butzengeiger S. (2019). Additionality revisited: guarding the integrity of market mechanisms under the Paris Agreement. Clim. Policy.

[33] Schäfer M., Herlev Gebara C., Bjørn A., Brander M. (2025). Identifying options for additionality tests in the context of scope 2 market-based accounting. Carbon Manag..

[34] Ever.green Additionality Methodology for High-impact RECs. https://www.ever.green/additionality.

[35] Giacobone B. (2024).

[36] GHG Protocol (2024). Overview of GHG Protocol Integration in Regulatory Climate Disclosure Rules. https://ghgprotocol.org/sites/default/files/2024-03/GHG-Protocol-Integration.pdf.

[37] European Commission (2022).

[38] Brander M., Bjørn A. (2023). Principles for accurate GHG inventories and options for market-based accounting. Int. J. Life Cycle Assess..

[39] Bjørn A., Lund J.F., Brander M. (2025). Up to half of companies would be behind on their climate targets under stricter scope 2 accounting rules. Environ. Res. Lett..

[40] U.S. Energy Information Administration Hourly Electric Grid Monitor. https://www.eia.gov/electricity/gridmonitor/dashboard/electric_overview/US48/US48.

[41] ENTSO-E Transparency Platform. https://transparency.entsoe.eu/.

[42] Standards Development and Governance Repository | GHG Protocol. https://ghgprotocol.org/standards-development-and-governance-repository.

[43] International Energy Agency (2023).

[44] Scope of the EU ETS - European Commission. https://climate.ec.europa.eu/eu-action/eu-emissions-trading-system-eu-ets/scope-eu-ets_en.

[45] Bruninx K., Moncada J.A., Ovaere M. (2022). Electrolytic hydrogen has to show its true colors. Joule.

[46] Vandyck T., Della Valle N., Temursho U., Weitzel M. (2023). EU climate action through an energy poverty lens. Sci. Rep..

[47] Fragkos P., Fragkiadakis K., Sovacool B., Paroussos L., Vrontisi Z., Charalampidis I. (2021). Equity implications of climate policy: Assessing the social and distributional impacts of emission reduction targets in the European Union. Energy.

[48] Bruninx K., Ovaere M. (2022). COVID-19, Green Deal and recovery plan permanently change emissions and prices in EU ETS Phase IV. Nat. Commun..

[49] Odenweller A., Ueckerdt F., Nemet G.F., Jensterle M., Luderer G. (2022). Probabilistic feasibility space of scaling up green hydrogen supply. Nat. Energy.

[50] Hoogsteyn A., Meus J., Bruninx K., Delarue E. (2024).

[51] European Commission (2022).

[52] German Federal Ministry for Economic Affairs and Energy (2020). The National Hydrogen Strategy. https://www.bmwk.de/Redaktion/EN/Publikationen/Energie/the-national-hydrogen-strategy.pdf?__blob=publicationFile&v=1.

[53] German Federal Ministry for Economic Affairs and Energy (2023). The National Hydrogen Strategy Update NHS. https://www.bmwk.de/Redaktion/EN/Publikationen/Energie/national-hydrogen-strategy-update.pdf?__blob=publicationFile&v=2.

[54] Golombek R., Irarrazabal A.A., Ma L. (2018). OPEC’s market power: An empirical dominant firm model for the oil market. Energy Econ..

[55] Egging R.G., Gabriel S.A. (2006). Examining market power in the European natural gas market. Energy Policy.

[56] Egging R., Holz F., Gabriel S.A. (2010). The World Gas Model: A multi-period mixed complementarity model for the global natural gas market. Energy.

[57] Ansari D. (2017). OPEC, Saudi Arabia, and the shale revolution: Insights from equilibrium modelling and oil politics. Energy Policy.

[58] De-León Almaraz S., Gelei A., Solymosi T. (2025). Coalition analysis for low-carbon hydrogen supply chains using cooperative game theory. Int. J. Hydrogen Energy.

[59] Wietschel M., Eckstein J., Riemer M., Zheng L., Lux B., Pieton N., Nolden C., Pfluger B., Thiel Z., Löschel A. (2021). https://publica-rest.fraunhofer.de/server/api/core/bitstreams/620b365f-d138-430d-94c8-f1d9eedaebe9/content.

[60] Hampp J., Düren M., Brown T. (2023). Import options for chemical energy carriers from renewable sources to Germany. PLoS One.

[61] Franzmann D., Heinrichs H., Lippkau F., Addanki T., Winkler C., Buchenberg P., Hamacher T., Blesl M., Linßen J., Stolten D. (2023). Green hydrogen cost-potentials for global trade. Int. J. Hydrogen Energy.

[62] Barner L. (2024). A multi-commodity partial equilibrium model of imperfect competition in future global hydrogen markets. Energy.

[63] Hauser E., Heib S., Hildebrand J., Rau I., Weber A., Welling J., Güldenberg J., Maaß C., Mundt J., Werner R. (2019). https://www.umweltbundesamt.de/sites/default/files/medien/1410/publikationen/2019-08-15_cc_30-2019_marktanalyse_oekostrom_ii.pdf.

[64] Q&A on simplification omnibus I and II. https://ec.europa.eu/commission/presscorner/detail/en/qanda_25_615.

